# F127-SE-tLAP thermosensitive hydrogel alleviates bleomycin-induced skin fibrosis via TGF-β/Smad pathway

**DOI:** 10.1186/s10020-024-00815-w

**Published:** 2024-04-19

**Authors:** Zhiqin Cao, Keke Zhang, Jingruo Liu, Yu Pan, Jiayi Shi, Luxin Li, Xiaocan Sun, Shiqi Li, Xiaohuan Yuan, Dan Wu

**Affiliations:** 1 Heilongjiang Province Key Laboratory of Anti-fibrosis Biotherapy, Mudanjiang Medical University, No. 3, Tongxiang Street, Aimin District, 157011 Mudanjiang, Heilongjiang China; 2 College of Life Sciences, Mudanjiang Medical University, 157011 Mudanjiang, Heilongjiang China

**Keywords:** Skin fibrosis, TGF-β1, LAP, SPACE, EMT

## Abstract

**Background:**

Skin fibrosis affects the normal function of the skin. TGF-β1 is a key cytokine that affects organ fibrosis. The latency-associated peptide (LAP) is essential for TGF-β1 activation. We previously constructed and prepared truncated LAP (tLAP), and confirmed that tLAP inhibited liver fibrosis by affecting TGF-β1. SPACE peptide has both transdermal and transmembrane functions. SPACE promotes the delivery of macromolecules through the stratum corneum into the dermis. This study aimed to alleviate skin fibrosis through the delivery of tLAP by SPACE.

**Methods:**

The SPACE-tLAP (SE-tLAP) recombinant plasmid was constructed. SE-tLAP was purified by nickel affinity chromatography. The effects of SE-tLAP on the proliferation, migration, and expression of fibrosis-related and inflammatory factors were evaluated in TGF-β1-induced NIH-3T3 cells. F127-SE-tLAP hydrogel was constructed by using F127 as a carrier to load SE-tLAP polypeptide. The degradation, drug release, and biocompatibility of F127-SE-tLAP were evaluated. Bleomycin was used to induce skin fibrosis in mice. HE, Masson, and immunohistochemistry were used to observe the skin histological characteristics.

**Results:**

SE-tLAP inhibited the proliferation, migration, and expression of fibrosis-related and inflammatory factors in NIH-3T3 cells. F127-SE-tLAP significantly reduced ECM production, collagen deposition, and fibrotic pathological changes, thereby alleviating skin fibrosis.

**Conclusion:**

F127-SE-tLAP could increase the transdermal delivery of LAP, reduce the production and deposition of ECM, inhibit the formation of dermal collagen fibers, and alleviate the progression of skin fibrosis. It may provide a new idea for the therapy of skin fibrosis.

## Background

Many physical and chemical factors can cause skin damage. Severe deposition of extracellular matrix in the dermis leads to delayed wound healing. Excessive deposition can lead to fibrosis and directly affect the normal structure and function of the skin (Wang et al. 2023). In addition, autoimmune diseases can lead to skin fibrosis.

Studies show that more than 100 million people are affected by skin fibrosis each year. A number of therapeutic strategies are available to prevent and reduce fibrosis formation, including surgical resection, radiation therapy, laser therapy, and drug injection therapy (Bukiri and Volkmann 2022). As the traditional treatment of fibrosis, single surgical treatment cannot achieve satisfactory results in the treatment of skin fibrosis (Zhong et al. 2024). Due to the inherent carcinogenic risk, radiation therapy is generally not considered for the treatment of skin fibrosis (Yang et al. 2020). In addition, nearly 90% patients with skin fibrosis who receive radiation therapy suffer from moderate to severe skin damage. In recent years, transdermal drug delivery (Chen et al. 2006) has become a well-established alternative route of drug delivery to avoid injectable damage and reduce the impact on other tissues and organs.

Skin lesions stimulate TGF-β1 synthesis and secretion and increase local extracellular stores of latent TGF-β1 (Vander Ark et al. 2018). It also stimulates wide signal transduction that promotes the release of active TGF-β1 from inactive complexes (Baral et al. 2021). Activated TGF-β1 regulates the cytokines and epithelial-mesenchymal transition (EMT), resulting in the increase of dermal connective tissue, which is also the main pathological manifestation of skin fibrosis (Eckes et al. 2014; Manetti et al. 2017). Macrophages are one of the important initial sources of TGF-β1(Frangogiannis 2020; Nolte and Margadant 2020; Peng et al. 2022). The activated TGF-β1 also further stimulates macrophages to play its fibrosis activity (Lee and Massagué 2022).

During the development of skin fibrosis, the number and type of cells have changed, and except for macrophages, the damaged area recruits a mass of fibroblasts and inflammatory cells(Eckes et al. 2014; Liu et al. 2014). Changes in the microenvironment caused by inflammation not only directly enhance the generation of collagen in fibroblasts, but also induce EMT at the injured area(Mack 2018). These changes promote the transformation of epithelial cells and fibroblasts into myofibroblasts that express α-smooth muscle actin (α-SMA) (Klingberg et al. 2013; Ji et al. 2016). The overexpression of α-SMA in the dermis increases the stress of ECM, which promotes the remodeling of extracellular matrix and fibrosis (Andrews et al. 2016). However, studies have shown that EMT is reversible (Pei et al. 2019). Block TGF-β1 signaling pathway is an effective way to prevent fibrosis progression, which can reduce the EMT-mediated expression of fibronectin and vimentin, and tissue remodeling. It can also down-regulate the proliferation, migration, and extracellular matrix secretion mediated by TGF-β1 (Chen et al. 2022).

At present, one of the effective ways to treat skin fibrosis is to block the TGF-β1 transduction pathway or block its binding with the TGF-β1 receptor (Li et al. 2021). LAP is essential for TGF-β1 activation. We previously constructed and prepared truncated tLAP, and confirmed that tLAP inhibits liver fibrosis by affecting TGF-β1 activation (Song et al. 2022; Qiu et al. 2023). In this study, we attempted to use tLAP to alleviate skin fibrosis. Combined with the characteristics of structure and function of skin tissue, a transdermal peptide SPACE was used to modify tLAP (Prausnitz and Langer 2008; Kumar et al. 2015). SPACE peptide is a peptide with both transdermal and transmembrane functions. SPACE peptide achieves the transmembrane (Nasrollahi et al. 2012) and transdermal delivery of peptides (Chen et al. 2014a) and siRNA (Liu et al. 2017), which can target the dermis. This study attempted to inhibit skin fibrosis through SPACE delivery of tLAP. Transdermal administration has become a mature alternative route of drug delivery. The F127(Kumar et al. 2015; Youn et al. 2021) thermosensitive hydrogels can be loaded with proteins, peptides, or small-molecule drugs. It is liquid at low temperatures and then forms a hydrogel at body temperature. It can slow the release of the drug, thereby prolonging its effects(Zhang et al. 2021). F127 was used as a carrier to load SE-tLAP, which avoided the damage caused by repeated injection, increased the availability and controlled release of polypeptides, and degraded spontaneously(Dantas Lopes Dos Santos et al. 2021; Zhang et al. 2021). In this study, a recombinant fusion polypeptide SE-tLAP was designed and purified. The thermosensitive F127-SE-tLAP hydrogel was developed to investigate its effect on skin fibrosis. It may provide a new idea for the therapy of skin fibrosis.

## Materials and methods

### Reagents and chemicals

IPTG was purchased from Gibco (Gand Island, NY, USA). Ni-agarose affinity chromatography columns were purchased from Invitrogen (Cambridge, UK). Bleomycin hydrochloride (BLM) was purchased from Maclean (Shanghai, China). DMEM was supplied by Gibco (Gand Island, NY, USA); Fetal bovine serum was purchased from PAN-Biotech (Heilbronn, Germany); Recombinant human TGF-β1 was purchased from PEPROTECH (Rocky Hill, USA); GAPDH, α-SMA and FN antibodies were purchased from Affinity (Cincinnati, OH, USA); Col-I ELISA kit was purchased from MEIMIAN (Jiangsu, China); Hydroxyproline kit was purchased from Nanjing Jiancheng (Nanjing, China).

### Plasmid construction

The linker (GGGGS) and SPACE peptide (ACTGSTQHQCG) sequences were added to the C-terminal of the tLAP sequence. The SE-tLAP sequences were inserted into the pet28a vector. SE-tLAP/pet28a recombinant plasmid was constructed.

### Expression, purification, and identification of SE-tLAP

The SE-tLAP recombinant plasmid was transformed into Rosetta (DE3) competent cells. Peptide expression was induced by IPTG. The bacteria were collected for ultrasonic crushing, and the supernatant was collected after high-speed centrifugation. The recombinant peptides were purified by Ni-agarose affinity columns and detected by SDS-PAGE. Purified SE-tLAP was identified by Western blot. The concentration of the anti-His antibody was 1:1000, and the concentration of the goat anti-mouse IgG antibody was 1:10000.

### Cell viability assay

The cell proliferation was investigated by conventional MTT assay. In short, NIH-3T3 cells (1 × 10^5^ cells/well) were inoculated into a 96-well plate. TGF-β1 (10 ng/mL) was added for 12 h. SE-tLAP (30, 60, 90, 120, 150 µg/mL) was added for 24 h. Then, 20µL MTT (5 mg/mL) was added for 4 h. Subsequently, the supernatant was removed and the formazan crystal was dissolved in DMSO (150 µL). The absorbance of 490 nm was detected by an enzyme-labeled instrument.

### Wound healing assay

NIH-3T3 cells (15 × 10^4^ cells/well) were inoculated into a 6-well plate and scratched. The cells were treated with TGF-β1 (10 ng/mL) and recombinant peptides. The results were observed by a microscope.

### Transwell migration assay

NIH-3T3 cell migration was determined using the Transwell Boyden chamber. The cells were cultured into a 24-well plate with serum-containing medium for 12 h, then treated with TGF-β1 (10 ng/mL) for 12 h, added the tLAP or SE-tLAP for 24 h. The non-migrating cells on the surface of Transwell membrane were washed with PBS, and the migrating cells were stained with 5% cresol violet for 15 min. The results were observed by a microscope.

### Real-time PCR

NIH-3T3 cells were inoculated and treated by TGF-β1(10 ng/mL) and recombinant peptides. Total RNA was extracted and reverse-transcribed. The gene expression was detected by the StepOne RT PCR system.

### Cell immunofluorescence

The cells were fixed with 4% paraformaldehyde for 20 min. Then the cells were infiltrated with 0.1% Triton X-100 and sealed with 5% BSA for 1 h. The cells were incubated overnight with anti-α-SMA (1:250) and anti-Col-I (1:250) at 4 °C, and then a fluorescent secondary antibody was applied for 1.5 h. Cells were re-stained with DAPI for 15 min. The results were observed using laser confocal microscopy.

### Western blot

NIH-3T3 cells were treated with TGF-β1 and polypeptides. The proteins were extracted with a lysate buffer and loaded by SDS-PAGE, and then transferred to PVDF membranes. The membrane was sealed in 5% skim milk for 2 h, rinsed, and incubated with the corresponding antibody at 4℃, followed by the secondary antibody at 37℃ for 1 h. ECL was used for color development.

### Enzyme-linked immunosorbent assay (ELISA)

NIH-3T3 cells were treated with TGF-β1 and polypeptides. The cells were digested with trypsin and supernatant was collected. After repeated freeze-thaw, the supernatant was centrifuged. The collagen I content was determined by the ELISA kit.

### Preparation of the hyrogels

0.6 g F127 was added into 3mL polypeptide solution and incubated at 4℃ for 12 h until completely dissolved. F127, F127-tLAP, and F127-SE-tLAP hydrogels with a concentration of 20% were prepared and refrigerated at 4℃.

### Detection of hydrogel degradation and drug release

1mL PBS was added into 0.5 g F127-tLAP and F127-SE-tLAP hydrogel and incubated at 37℃. The hydrogel weight and polypeptide content in PBS were measured every 4 h.

### Hydrogel biocompatibility test

Biocompatibility of the hydrogel was assessed by double staining with Calence AM and PI. NIH-3T3 cells were inoculated in a 6-well plate. 100 µL hydrogel was added to each well at 37 ℃ and 5% CO_2_ for 24 h. The cells were stained with Calence AM and PI for 15 min. The results were observed by fluorescence microscope.

### Animal experiments

The animal experiments were approved by the Institutional Animal Care and Use Committee of Mudanjiang Medical University. ICR mice weighing 23 ± 2 g, were allowed to adapt at a constant temperature (23 ± 2℃) for 7 days before use. The mice were randomly divided into the control group, BLM group, F127 group, F127-tLAP group, and F127-SE-tLAP group. The skin fibrosis model was established by subcutaneous injection of 100µL bleomycin (1 mg/mL). The injection was given every 2 days for 4 weeks. After 2 weeks of injection, 100 µL hydrogel containing 30 µg peptide was applied to the mice once every 2 days for 2 weeks.

### Transdermal test of hydrogel

ICR mice were randomly divided into two groups. The hydrogel was prepared after labeling tLAP and SE-tLAP with FITC. 100 µL fluorescent-labeled hydrogel containing 30 ug polypeptide was applied to the back of mice. The skin tissue was embedded in OCT and sliced on a cryostat. Fluorescence was detected using confocal microscopy (Aoki et al. 2019).

### Pathological staining and immunohistochemistry

The skin tissues were fixed with 4% formaldehyde, dehydrated, and embedded. Routinely prepared in paraffin sections with a thickness of 5 μm. Paraffin sections were stained and observed by an optical microscope. The paraffin sections for immunohistochemistry were 4 μm thick. Sections were incubated with α-SMA and Col-I antibody at 4℃, followed by the secondary antibody at 37℃ for 1 h. After staining with DAB, the sections were counterstained with hematoxylin. The sections were sealed in a neutral resin and then investigated by an optical microscope.

### Detection of hydroxyproline

The skin tissue was weighed and chopped, and PH of its hydrolysate was adjusted to 6.0-6.8. The sample was centrifugally filtered and adjusted concentration with PBS. The absorbance value was detected at 550 nm using a hydroxyproline content detection kit.

### Statistical analysis

Experimental data were analyzed by GraphPad Prism 8.3. The results were expressed as mean ± standard deviation (x ± s). Groups were compared by one-way analysis of variance. *P* < 0.05 represented statistically significant.

## Results

### Construction, preparation, and identification of SE-tLAP

**Fig. 1:**
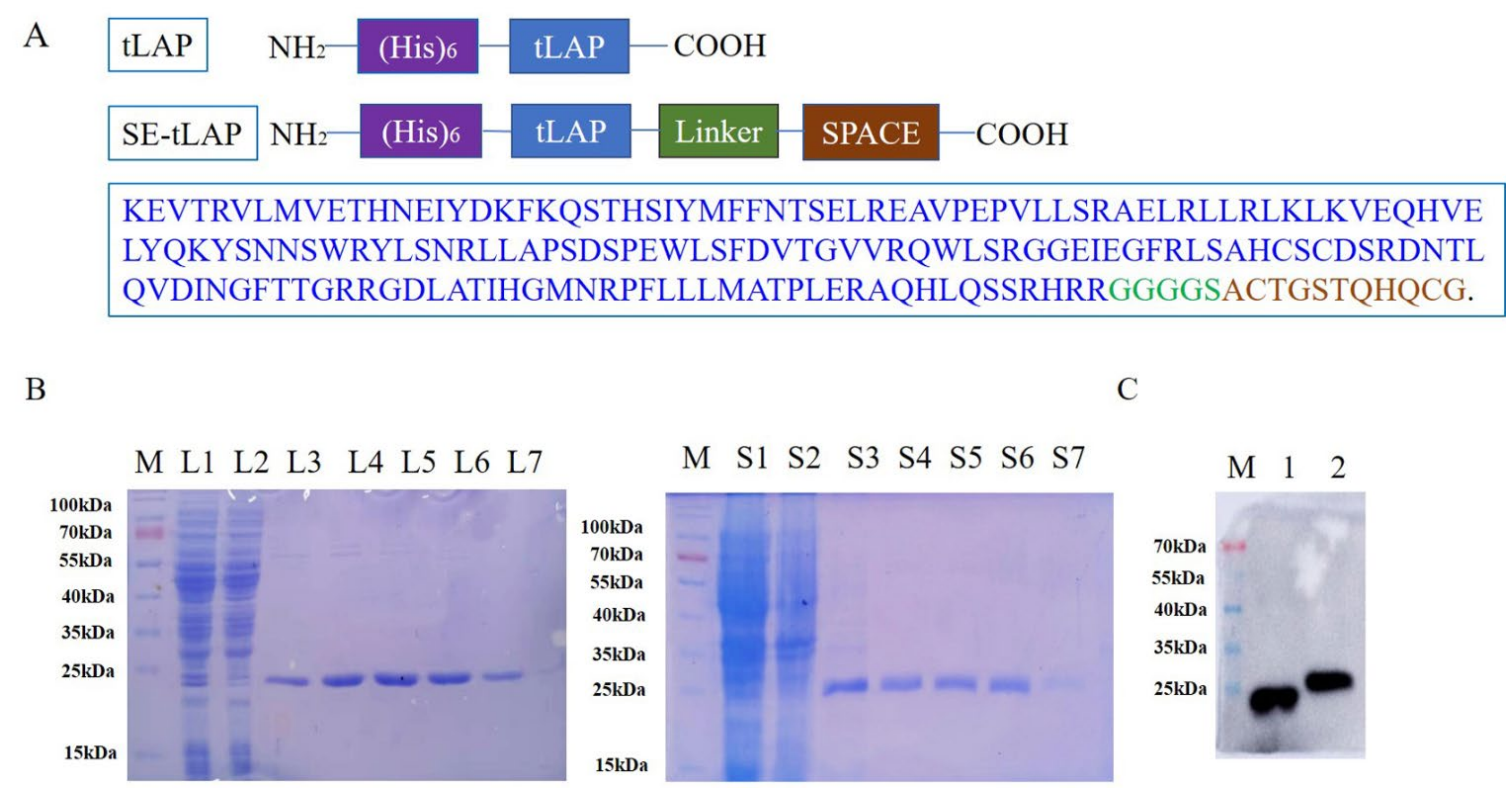
Plasmid construction, purification, and identification of SE-tLAP. (**A**). Composition of SE-tLAP sequence; (**B**). Purification of tLAP and SE-tLAP. M, Marker; L1-L7, tLAP; S1-S7, SE-tLAP; 1, Crushing bacterial solution, 2, Supernatant, 3–7, Elution; (**C**). Expression identification of tLAP and SE-tLAP. M, Marker; 1, tLAP; 2. SE-tLAP

SE-tLAP sequence was cloned and inserted into the pet28a vector to construct SE-tLAP/pet28a recombinant plasmid (Fig. 1A). The expression condition of SE-tLAP was cultured at 37℃ to OD600 value of 0.8, and induced by 0.3mM IPTG at 16℃ for 16 h. The polypeptides containing histidine tag were purified by Nickel affinity chromatography. The purification results showed that the expression level of SE-tLAP was not affected by the modification of the SPACE peptide (Fig. 1B). SE-tLAP was obtained by imidazole elution and identified by Western blot (Fig. 1C).

### SE-tLAP inhibits TGF-β1-induced proliferation and migration of NIH-3T3 cells

**Fig. 2:**
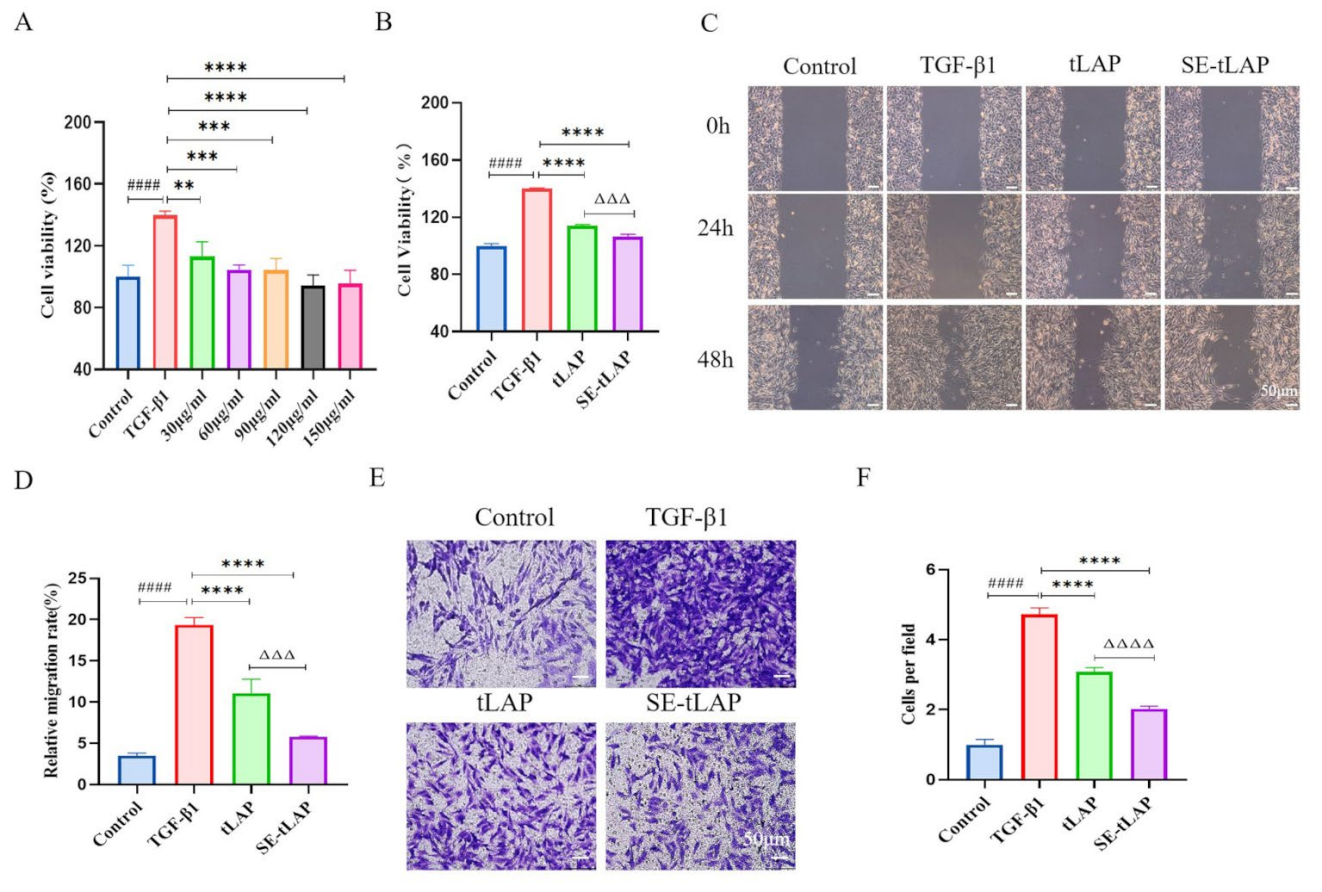
Effects of tLAP and SE-tLAP on the proliferation and migration of TGF-β1-induced NIH-3T3 cell. (**A**). Determination of the optimal concentration of SE-tLAP. (**B**). The effects of tLAP and SE-tLAP on NIH-3T3 cell proliferation. (**C**). Detection of cell migration by Wound healing assay, magnifcation×200, scale bars = 50 μm. (**D**). Statistical analysis of the results of Wound healing assay. (**E**). Detection of cell migration by Transwell assay, magnifcation×200, scale bars = 50 μm. (**F**). Statistical analysis of the results of Transwell assay. ^####^*P* < 0.0001 vs. Control; **P < 0.01, ***P < 0.001, ****P < 0.0001 vs. TGF-β1; ^ΔΔΔ^*P* < 0.001, ^ΔΔΔΔ^*P* < 0.0001, tLAP vs. SE-tLAP

The effects of tLAP and SE-tLAP on TGF-β1-induced cell proliferation were evaluated by MTT. Compared with the control, TGF-β1 significantly induced cell proliferation. 30, 60, 90, 120, and 150 µg/ml SE-tLAP can inhibit TGF-β-induced cell proliferation (Fig. 2A). A concentration of 60 µg/mL was chosen for subsequent experiments. The results showed that SE-tLAP had a better inhibitory effect on cell proliferation than tLAP, which may be due to the high permeability and transmembrane functions of SPACE (Fig. 2B). The wound healing assay was used to investigate the effect of recombinant peptides on cell migration. The results showed that TGF-β1 significantly induced cell migration, while the tLAP and SE-tLAP groups inhibited cell migration (Fig. 2C). The cell migration ratio of the SE-tLAP group was lower than that of the tLAP group (Fig. 2D). Transwell results showed that the number of cells in the tLAP and SE-tLAP groups was reduced compared with that in the TGF-β1 group (Fig. 2E). Both tLAP and SE-tLAP inhibited TGF-β1-induced cell migration, and the inhibition was more significant in the SE-tLAP group (Fig. 2F). These results suggested that SE-tLAP was more effective than tLAP in inhibiting TGF-β1-induced cell proliferation and migration.

### SE-tLAP inhibits TGF-β1-induced inflammation and fibrosis in NIH-3T3 cells

**Fig. 3:**
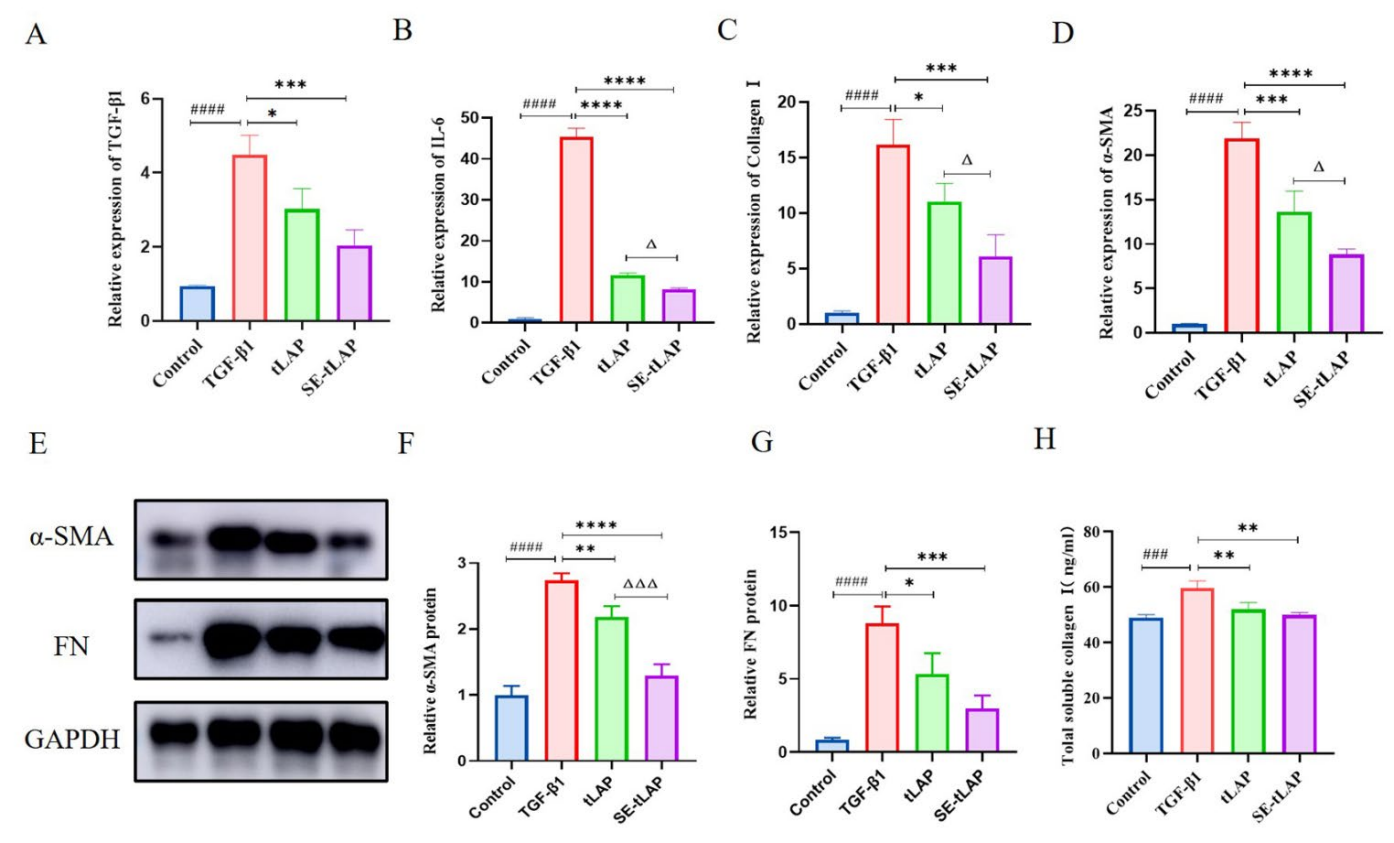
Effects of tLAP and SE-tLAP on the inflammation and fibrosis of TGF-β1-induced NIH-3T3 cells. (**A**-**D**). Detection of the mRNA expressions of TGF-β1, IL-6, α-SMA, and Col-I in NIH-3T3 cells. (**E**). Protein expressions of α-SMA and FN in NIH-3T3 cells. (**F**-**G**). Quantitative analysis of α-SMA and FN. (**H**). Detection of the Col-I expression in NIH-3T3 cells. ^###^*P* < 0.001, ^####^*P* < 0.0001 vs. Control; *P < 0.05,**P < 0.01, ***P < 0.001, ****P < 0.0001 vs. TGF-β1; ^Δ^*P* < 0.05,^ΔΔΔ^*P* < 0.001, tLAP vs. SE-tLAP

The mRNA expression of inflammatory and fibrosis-related factors was investigated by RT-qPCR. The expression of TGF-β1 was significantly increased in the model group. tLAP and SE-tLAP inhibited the expression, and the SE-tLAP group had a better inhibitory effect (Fig. 3A). TGF-β1 induced inflammation in NIH-3T3 cells. Compared with the control, the expression of IL-6 was remarkably increased in the TGF-β1 group, and the expression of IL-6 in the tLAP and SE-tLAP groups was significantly decreased (Fig. 3B). In addition, TGF-β1 promotes the production of collagen fibers in fibroblasts, while stimulating fibroblasts to develop EMT and transform into myofibroblasts that overexpress α-SMA. The expression of Col-I and α-SMA was obviously increased in the TGF-β1 group, and tLAP and SE-tLAP inhibited the overexpression, while SE-tLAP had a better inhibition effect (Fig. 3C, D).

**Fig. 4:**
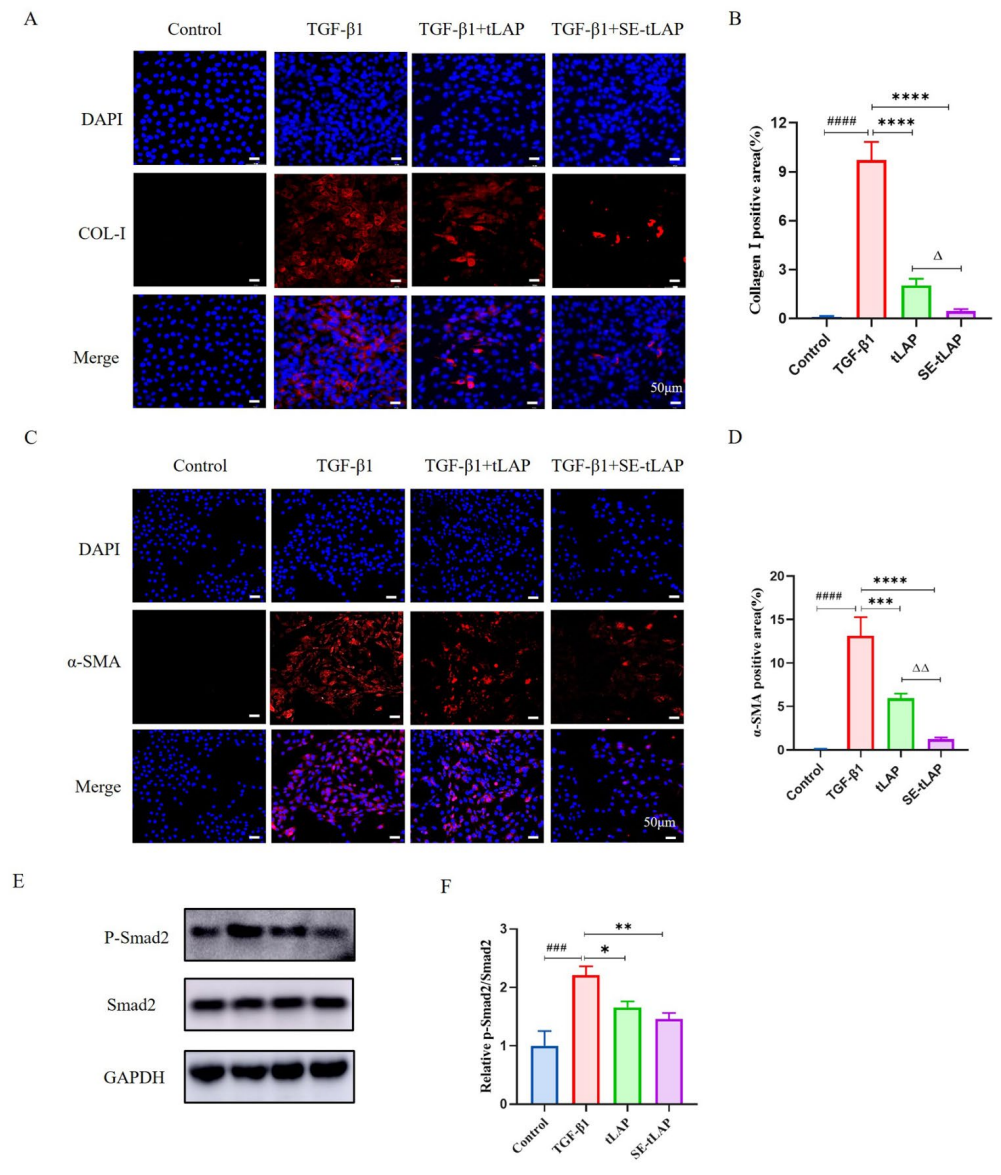
Detection of the expression of Col-I and α-SMA in NIH-3T3 cells by immunofluorescence. (**A**). Immunofluorescence detection of Col-I (magnifcation×200, scale bars = 50 μm). DAPI (blue, nuclear staining), Col-I (red). Magnifcation×200, scale bars = 50 μm. (**B**). Fluorescence analysis of Col-I. (**C**). Immunofluorescence detection of α-SMA. (magnifcation×200, scale bars = 50 μm). DAPI (blue, nuclear staining), α-SMA (red). (**D**). Fluorescence analysis of α-SMA. (**E**). Protein expressions of Smad2 and P-Smad2 in NIH-3T3 cells. (**F**). Quantitative analysis of P-Smad2. ^###^*P* < 0.001, ^####^*P* < 0.0001 vs. Control; *P < 0.05,**P < 0.01,****P < 0.0001 vs. TGF-β1; ^Δ^*P* < 0.05, ^ΔΔΔΔ^*P* < 0.0001, tLAP vs. SE-tLAP

Cell immunofluorescence, Western blot, and ELISA were used to investigate the protein expression of fibrosis-related and inflammatory factors in NIH-3T3 cells. The expression of α-SMA and FN was up-regulated by TGF-β1, while tLAP and SE-tLAP down-regulated the expression, and SE-tLAP had a better inhibitory effect (Fig. 3E, F, G). The expression of Col-I was detected by ELISA. TGF-β1 promoted the expression of Col-I, while tLAP and SE-tLAP significantly decreased the expression of Col-I, and SE-tLAP had a better inhibitory effect (Fig. 3H). The fluorescence intensity of α-SMA and Col-I in the TGF-β1 group was significantly enhanced than that in the control group, indicating that the expression of α-SMA and Col-I was increased under the induction of TGF-β1, while their expression was inhibited by tLAP and SE-tLAP, and SE-tLAP was more effective (Fig. 4A-D). The above results suggested that SE-tLAP inhibited the expression of fibrosis-related and inflammatory factors in TGF-β1-induced NIH-3T3 cells.

### SE-tLAP inhibits TGF-β1-induced fibrosis in NIH-3T3 cells via TGF-β/Smad pathway

The expression of multiple signal molecules has changed in skin fibrosis, thus affecting the signaling pathways in which they participate. The activation of TGF-β promotes the phosphorylation of signal molecules under the TGF-β/Smad pathway. The PCR results showed that SE-tLAP could effectively inhibit the expression of TGF-β1. Western blot results showed that TGF-β1 significantly stimulated the phosphorylation of Smad2 in NIH-3T3 cells, and SE-tLAP reduced the phosphorylation of Smad2 by inhibiting TGF-β1(Fig. 4E, F). The results indicated that SE-tLAP could inhibit TGF-β1-induced NIH-3T3 cell fibrosis via TGF-β/Smad pathway.

### Preparation and characterization of F127-SE-tLAP hydrogel

**Fig. 5:**
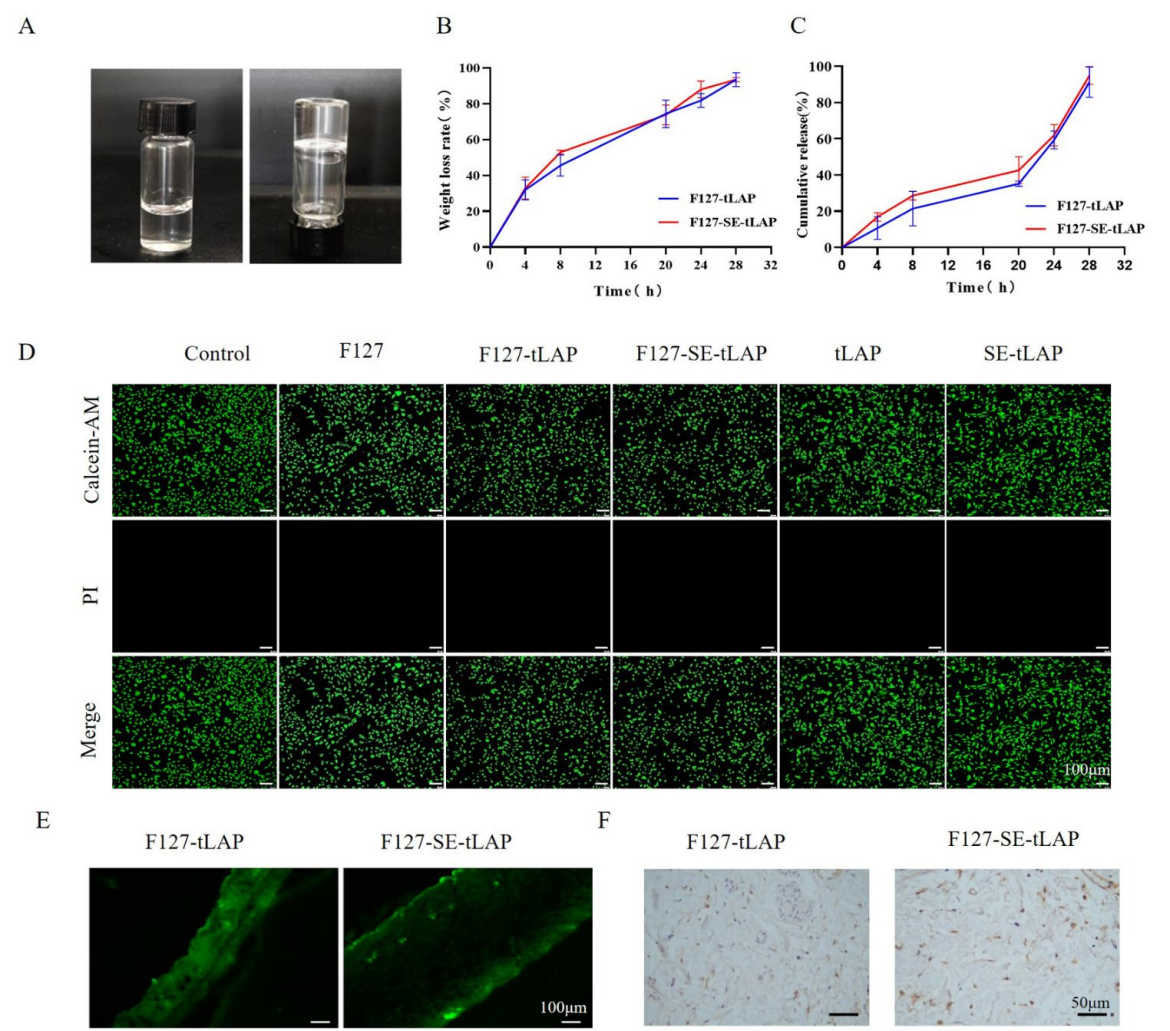
Preparation and characterization of F127-SE-tLAP hydrogel. (**A**). F127-SE-tLAP is liquid at low temperatures and forms a hydrogel above 25 °C. (**B**). Degradation of F127-tLAP and F127-SE-tLAP hydrogel. (**C**). Released curve of F127-tLAP and F127-SE-tLAP hydrogel. (**D**). Biocompatibility testing of F127-tLAP and F127-SE-tLAP (magnifcation×100, scale bars = 100 μm). Calcein-AM (green, living cells). PI (Red, dead cells). (**E**). Transdermal detection of F127-tLAP and F127-SE-tLAP hydrogel. (magnifcation×100, scale bars = 100 μm). (**F**). Immunohistochemical staining of anti-His (magnifcation×400, scale bars = 50 μm)

F127-SE-tLAP hydrogel is liquid at low temperatures and rapidly transforms into a hydrogel above 25 °C (Fig. 5A). In order to evaluate the degradation and drug release performance of F127-SE-tLAP hydrogels, the release of polypeptides and the weight loss of hydrogels were investigated at different time points at 37℃. F127-tLAP and F127-SE-tLAP hydrogels degraded about 70% after 20 h. With the degradation of the hydrogel, the peptides are gradually released. The release rate of F127-tLAP and F127-SE-tLAP reached 60% after 24 h, and the gel degradation and release of F127-SE-TLAP reached nearly 100% after 28 h (Fig. 5B, C). The results suggested that F127-tLAP and F127-SE-tLAP hydrogels had favorable degradation and drug slow-release properties.

In addition, the biocompatibility of F127-tLAP, and F127-SE-tLAP hydrogels was detected by Calcein-AM/PI staining. The results showed that there was no statistical difference in the intensity of green fluorescence of labeled live cells, and no red fluorescence of labeled dead cells was detected among all groups, suggesting that F127-tLAP and F127-SE-tLAP hydrogels had favorable biocompatibility (Fig. 5D).

### Transdermal properties of F127-SE-tLAP hydrogel

Fluorescence-labeled F127-tLAP and F127-SE-tLAP hydrogels were applied to the back skin of mice for 12 h, and the results showed that the transdermal effect of F127-SE-tLAP hydrogel was stronger than that of F127-tLAP (Fig. 5E). The His labeled immunohistochemical staining of skin tissue showed more peptide release in the skin of the F127-SE-tLAP group (Fig. 5F). These results suggested that F127-SE-tLAP hydrogel had better transdermal properties.

### F127-SE-tLAP alleviates BLM-Induced histopathological changes in mice

**Fig. 6:**
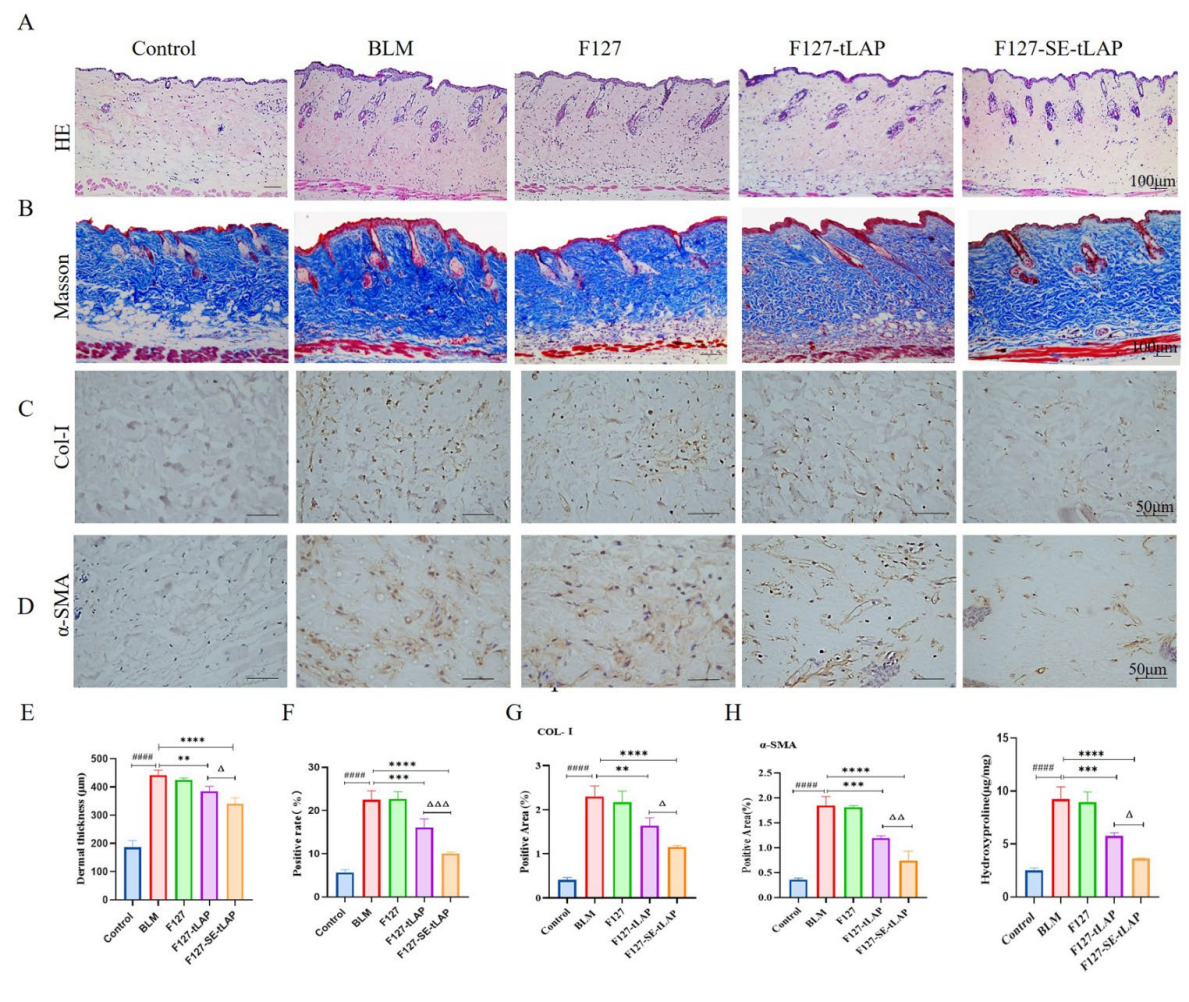
6F127-SE-tLAP hydrogel alleviated bleomycin-induced skin fibrosis. (**A**). HE staining results of skin tissues (Magnifcation×100, scale bars = 100 μm). (**B**). Masson staining results of skin tissues (Magnifcation×100, scale bars = 100 μm). (**C**). Immunohistochemical staining of Col-I (magnifcation×400, scale bars = 50 μm). (**D**). Immunohistochemical staining of α-SMA (magnifcation×400, scale bars = 50 μm). (**E**). Analysis of the dermal thickness. (**F**). Quantitative analysis of Masson. (**G**). Immunohistochemical analysis of Col-I. (**H**). Immunohistochemical analysis of α-SMA. (**I**). Analysis of hydroxyproline content in skin tissues. ^####^*P* < 0.0001 vs. Control; **P < 0.01,***P < 0.001, ****P < 0.0001 vs. TGF-β1; ^Δ^*P* < 0.05, ^ΔΔ^*P* < 0.01, ^ΔΔΔ^*P* < 0.001,F127-tLAP vs. F127-SE-tLAP

The skin fibrosis model was induced by subcutaneous injection of BLM. After BLM injection, the skin on the back developed keratosis and thickening. The skin became hard and less elastic, and the hair growth in the injection area was slow. The effects of F127-tLAP and F127-SE-tLAP on BLM-induced skin pathological changes in mice were evaluated by pathological staining (Fig. 6A, B). The expression of Col-I and α-SMA in skin tissue was investigated by immunohistochemistry (Fig. 6C, D). The results showed that F127-SE-tLAP significantly inhibited BLM-induced dermal thickening (Fig. 6E). Compared with the control, the inflammatory cell infiltration and collagen deposition were increased in the BLM group, and the collagen fibers were closely distributed and disordered. Compared with the BLM group, the inflammatory cell infiltration and collagen fiber hyperplasia in the F127-tLAP and F127-SE-tLAP groups were significantly reduced, and the fibers were neatly arranged (Fig. 6F). Col-I is the main component of the extracellular matrix in the dermis and is overexpressed in skin fibrosis. Compared with the control, the expressions of Col-I and α-SMA were increased in the BLM group. The overexpression of Col-I and α-SMA was inhibited in F127-tLAP and F127-SE-tLAP groups (Fig. 6G, H). These results suggested that F127-tLAP and F127-SE-tLAP alleviated the pathological changes of BLM-induced skin fibrosis, and F127-SE-tLAP was more effective than F127-tLAP.

### Effect of F127-SE-tLAP on hydroxyproline content in skin tissue

The content of Hydroxyproline (HYP) in tissues is an important index of the degree of skin fibrosis and reflects the collagen metabolism of skin tissues. The HYP content of skin tissue in the BLM group was significantly increased. Compared with the BLM group, the content of hydroxyproline in the F127-tLAP and F127-SE-tLAP groups was significantly decreased, suggesting that collagen proliferation was inhibited. The collagen inhibition was more significant in the F127-SE-tLAP group (Fig. 6I). The histopathological and biochemical results indicated that F127-SE-tLAP could alleviate BLM-induced skin fibrosis in mice.

## Discussion

Skin fibrosis is a complicated pathological process. In the analysis of the treatment of skin fibrosis by steroid injection and 5-FU at the focal site, it was learned nflammation is not the only critical step in the development of the fibrotic response. Inhibiting inflammation alone cannot effectively treat fibrotic hyperplasia(Gao et al. 2024). TGF-β is associated with inflammatory response and activation of fibrotic expression at the lesion site, and its activation plays an important role in the process of skin fibrosis. Blocking TGF-β and its signaling pathway is a good choice for the treatment of fibrosis(Ong et al. 2021). LAP is a key component of TGF-β activation. We previously designed a truncated LAP and confirmed that tLAP can interfere with TGF-β activation and inhibit its downstream pathway, thus alleviating liver fibrosis. SPACE peptide is a peptide with the dual function of transdermal and transmembrane(Nasrollahi et al. 2012; Chen et al. 2014b). Studies have shown that SPACE peptides can achieve transmembrane and transdermal delivery of polypeptides and siRNA, which can target drug delivery to the dermis(Liu et al. 2017).

In this study, we attempted to alleviate skin fibrosis by modifying the transdermal peptide SPACE to deliver tLAP.

Transdermal administration (Chen et al. 2006) has become a mature alternative route of drug delivery to avoid injectable damage and reduce the impact on other tissues and organs. In recent years, high loading efficiency hydrogels have garnered significant attention in the treatment of skin diseases due to the excellent biodegradability and stable rheological properties. The application of non-toxic hydrogels as carriers of dermatological drugs is a simple and effective strategy for the treatment of skin diseases. avoiding secondary skin trauma. The novel glycopeptide hydrogel prepared for chronic diabetic wound repair can achieve the stage release of MF and DS, and get a remarkable therapeutic effects (Wu et al. 2022). It has been documented that hydrogels as an ideal carrier system for deliver platelet-derived exosomes, mediate the production of hyaluronic acid and collagen, and decrease skin melanin pigmentation (Saberian and Abak 2024). Hydrogels have also been used for RNA delivery in RNA therapy (Wang and Burdick 2017). Studies have shown the ability of drug-loaded hydrogels to achieve controlled release while improving the therapeutic effect (Yu et al. 2020). F127 thermosensitive hydrogel is commonly used as a drug carrier. It can be loaded with proteins, peptides, or small molecules of drugs. It is liquid at low temperatures, and then at body temperature to form a hydrogel. It can make the drug slow release, thereby prolonging its efficacy. This temperature-sensitive hydrogel can be used for applications such as eye drops, local drug delivery, tumor treatment, and wound healing (Akash et al. 2012).

In this study, SE-tLAP was loaded with thermosensitive hydrogel F127, which avoided skin damage caused by repeated injection and increased the availability and controlled release of polypeptides. In vitro experiments, we observed that TGF-β induced the activation of inflammatory factor IL-6, stimulated the excessive proliferation of NIH-3T3 cells, promoted the transformation of fibroblasts into myofibroblasts, and mediated the deposition of collagen fibers and ECM. SE-tLAP and tLAP inhibited proliferation, migration, and expression of inflammatory and fibrosis-related factors in NIH-3T3 cells.

In this study, we selected the commonly used BLM-induced skin fibrosis, and further studies are needed on other skin fibrosis models under different mechanisms, such as hypochlorite induced immunodeficient skin fibrosis models.In BLM-induced skin fibrosis mice, TGF-β1 promoted collagen synthesis and ECM deposition in the dermis. F127-SE-tLAP inhibited the expression of inflammatory and fibrosis-related factors, alleviated the pathological changes of BLM-induced skin fibrosis, and inhibited the increase of hydroxyproline. In summary, F127-SE-tLAP hydrogel could alleviate bleomycin-induced skin fibrosis in mice.

## Conclusions

In this study, a recombinant fusion polypeptide SE-tLAP was designed and purified. SE-tLAP inhibited proliferation, migration and expression of fibrotic and inflammation-related factors in NIH-3T3 cells. The thermosensitive F127-SE-tLAP hydrogel was developed to investigate its effect on skin fibrosis. F127-SE-tLAP could increase the transdermal delivery of LAP, reduce the production and deposition of ECM, inhibit the formation of dermal collagen fibers, and alleviate the progression of skin fibrosis via TGF-β/Smad pathway. It may provide a new idea for the therapy of skin fibrosis.

## Data Availability

The data used and analysed during the current study are available from the corresponding author on reasonable request.

## Abbreviations

TGF-β1: Transforming growth factor-β1
LAP: latency-associated peptide
tLAP: truncated LAP
RT-qPCR: real-time quantitative PCR
ECM: extracellular matrix
SiRNA: small interfering RNA
SE-tLAP: SPACE-tLAP
