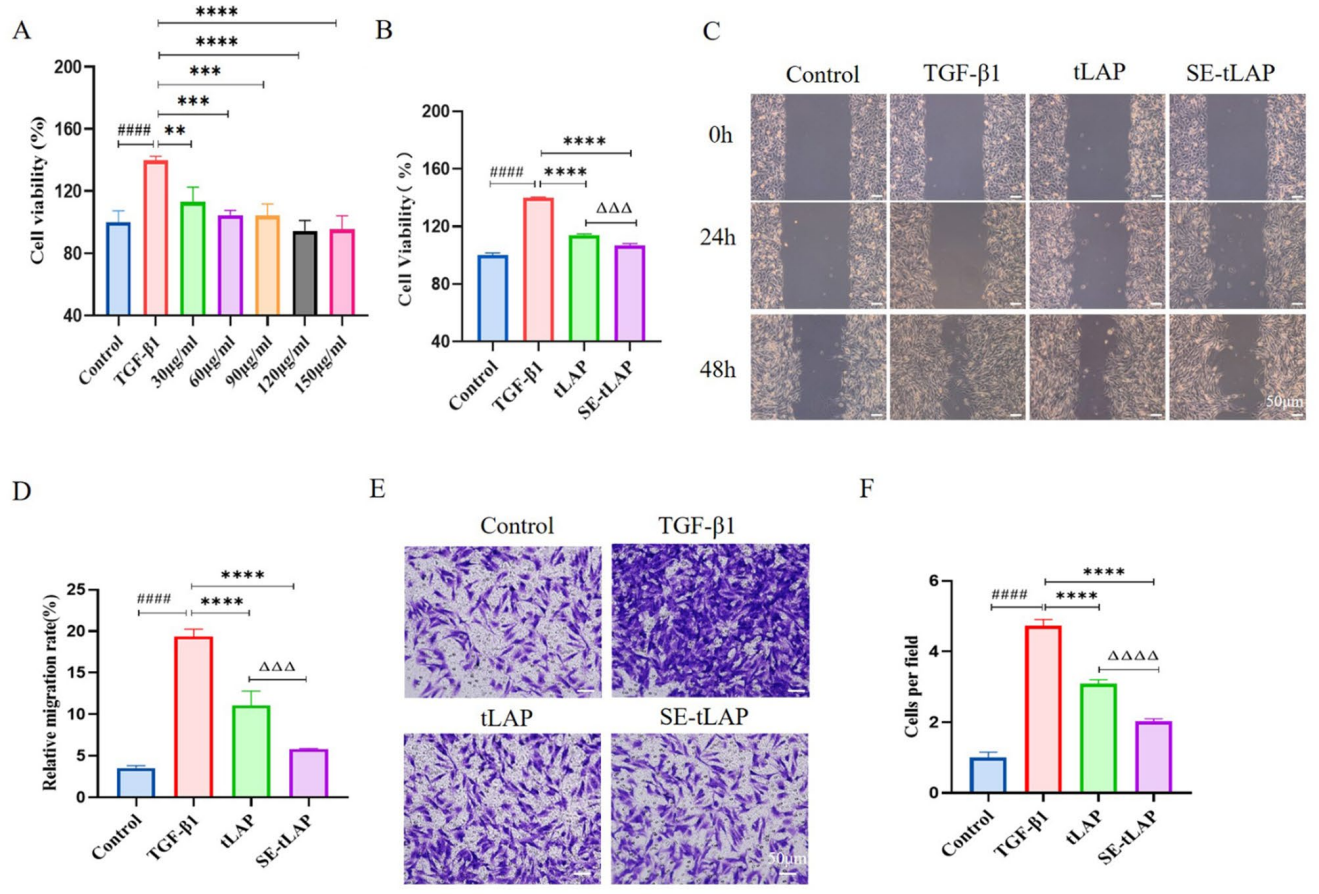# Correction: F127-SE-tLAP thermosensitive hydrogel alleviates bleomycin-induced skin fibrosis via TGF-β/Smad pathway

**DOI:** 10.1186/s10020-025-01291-6

**Published:** 2025-06-18

**Authors:** Zhiqin Cao, Keke Zhang, Jingruo Liu, Yu Pan, Jiayi Shi, Luxin Li, Xiaocan Sun, Shiqi Li, Xiaohuan Yuan, Dan Wu

**Affiliations:** 1 Heilongjiang Province Key Laboratory of Anti-fibrosis Biotherapy, Mudanjiang Medical University, No. 3, Tongxiang Street, Aimin District, Mudanjiang, Heilongjiang 157011 China; 2 College of Life Sciences, Mudanjiang Medical University, Mudanjiang, Heilongjiang 157011 China

**Correction: Mol Med (2024) 30:52**

10.1186/s10020-24-00815-w

In this article (Cao et al. 2024), Fig. 2 appeared incorrectly and have now been corrected in the original publication. For completeness and transparency, the old incorrect versions are displayed below.

Incorrect Fig. 2.
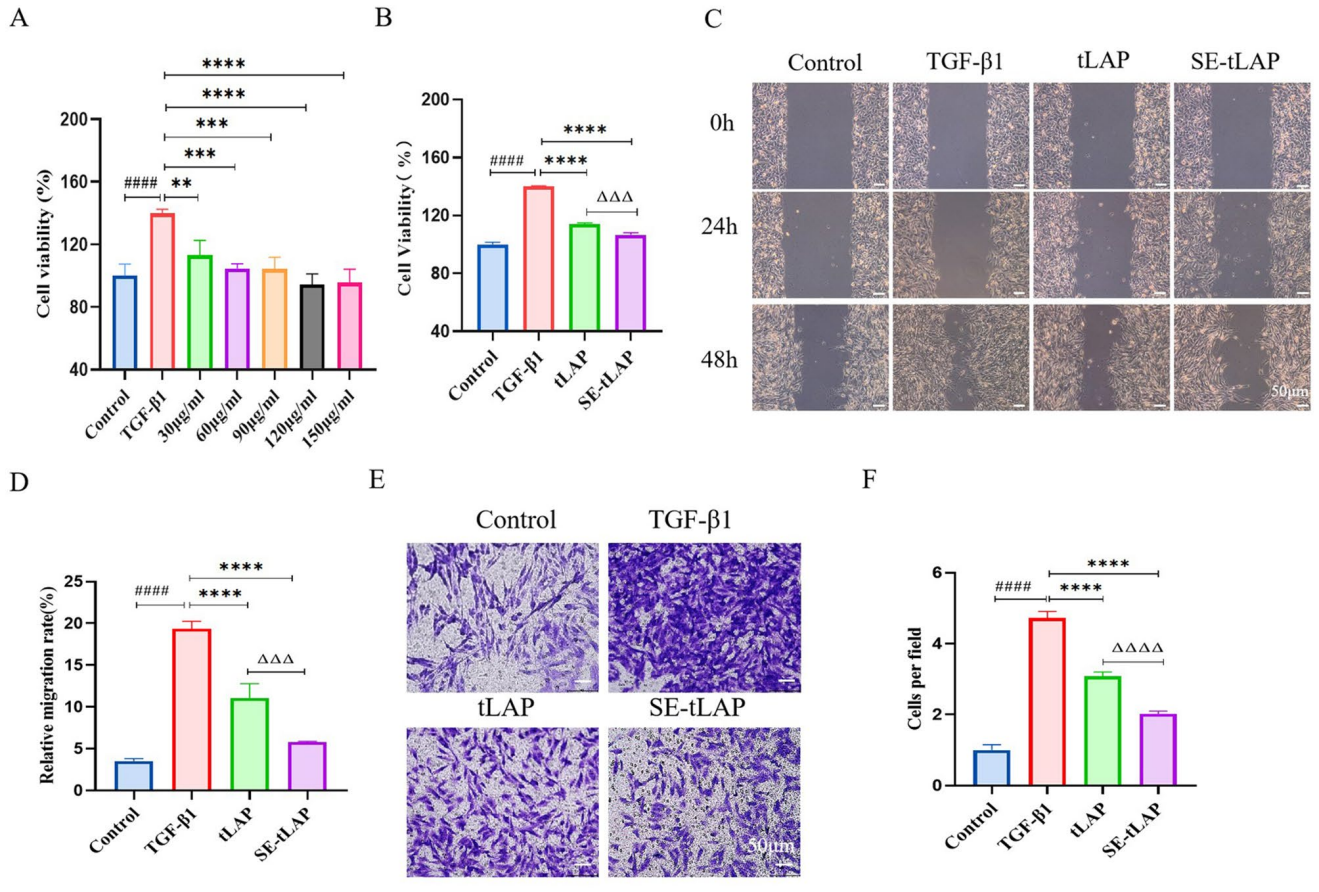


Correct Fig. 2.